# White matter alterations associated with chronic cannabis use disorder: a structural network and fixel-based analysis

**DOI:** 10.1038/s41398-024-03150-0

**Published:** 2024-10-11

**Authors:** Suzan Maleki, Joshua Hendrikse, Karyn Richardson, Rebecca A. Segrave, Sam Hughes, Edouard Kayayan, Stuart Oldham, Warda Syeda, James P. Coxon, Karen Caeyenberghs, Juan F. Domínguez D, Nadia Solowij, Dan I. Lubman, Chao Suo, Murat Yücel

**Affiliations:** 1 BrainPark, Turner Institute for Brain and Mental Health, School of Psychological Sciences and Monash Biomedical Imaging Facility, Monash University, Clayton, VIC Australia; 2 Movement and Exercise Neuroscience Laboratory, School of Psychological Sciences and Monash Biomedical Imaging Facility, Monash University, Clayton, VIC Australia; 3 Developmental Imaging, Murdoch Children’s Research Institute, Melbourne, VIC Australia; 4 Melbourne Brain Centre Imaging Unit, Department of Radiology, The University of Melbourne, Parkville, VIC Australia; 5 Cognitive Neuroscience Unit, School of Psychology, Deakin University, Burwood, VIC Australia; 6 School of Psychology, University of Wollongong, Wollongong, NSW Australia; 7 Turning Point, Eastern Health, Melbourne, VIC Australia; 8 Monash Addiction Research Centre, Eastern Health Clinical School, Monash University, Clayton, VIC Australia; 9 Neuroscience of Addiction and Mental Health Program, Healthy Brain and Mind Research Centre, School of Behavioral and Health Sciences, Faculty of Health Sciences, Australian Catholic University, Fitzroy, VIC Australia; 10 QIMR Berghofer Medical Research Institute, Herston, QLD Australia

**Keywords:** Addiction, Molecular neuroscience

## Abstract

Cannabis use disorder (CUD) is associated with adverse mental health effects, as well as social and cognitive impairment. Given prevalence rates of CUD are increasing, there is considerable efforts, and need, to identify prognostic markers which may aid in minimising any harm associated with this condition. Previous neuroimaging studies have revealed changes in white matter (WM) organization in people with CUD, though, the findings are mixed. In this study, we applied MRI-based analysis techniques that offer complimentary mechanistic insights, i.e., a connectome approach and fixel-based analysis (FBA) to investigate properties of individual WM fibre populations and their microstructure across the entire brain, providing a highly sensitive approach to detect subtle changes and overcome limitations of previous diffusion models. We compared 56 individuals with CUD (median age 25 years) to a sample of 38 healthy individuals (median age 31.5 years). Compared to controls, those with CUD had significantly increased structural connectivity strength (FDR corrected) across 9 edges between the right parietal cortex and several cortical and subcortical regions, including left orbitofrontal, left temporal pole, and left hippocampus and putamen. Utilizing FBA, WM density was significantly higher in those with CUD (FWE-corrected) across the splenium of the corpus callosum, and lower in the bilateral cingulum and right cerebellum. We observed significant correlation between cannabis use over the past month and connectivity strength of the frontoparietal edge, and between age of regular use and WM density of the bilateral cingulum and right cerebellum. Our findings enhance the understanding of WM architecture alterations associated with CUD.

## Introduction

With a shift towards legalisation and decriminalisation of recreational cannabis use, the prevalence of cannabis use disorder (CUD) appears to be increasing [1]. CUD is associated with adverse effects on mental health, including increased risk of mood and anxiety disorders, cognitive dysfunction, and social impairment [2]. As such, there has been considerable focus in recent years on assessing prognostic markers of CUD, which may aid in understanding and optimising long-term clinical outcomes [3]. Numerous structural MRI studies have investigated CUD-induced cortical and subcortical changes, yet evidence remains inconsistent with a meta-analysis revealing that about 50% of studies reported no significant grey matter (GM) alterations [3, 4]. Among significant findings, morphological alterations have been observed across the hippocampus [5–7], frontal cortex [8–11], and amygdala [7, 12, 13]. Besides morphological changes, CUD has also been linked to alterations in brain WM organization [14]. Previous diffusion MRI studies have identified heterogeneous alterations in fractional anisotropy (FA; a measure of WM integrity) across the corpus callosum [15–17], frontal regions [6, 16], cingulum [18, 19], and cerebellum [20]. Plausibly, some of the variability may be attributable to small sample sizes or variations in sample demographics (e.g., inclusion of comorbid diagnoses or polysubstance use, and discrepancy in frequency of use) [14].

In addition to demographic variability, the mixed findings may be explained by the employed model. Specifically, most of the previous studies assessing WM changes have employed the diffusion tensor imaging (DTI) model which is known to be limited in its capacity to identify intricate and diverse WM changes and therefore may contribute to inconsistent outcomes across studies [3, 21]. Moreover, the measure of FA lacks the microstructural specificity to fully characterise the organization of the white matter tracts. As such, advanced diffusion models using the FBA framework can help reconcile these discrepancies by measuring morphological information from both microscopic fibre density (FD) and macroscopic fibre cross-section (FC, the calibre of a fibre bundle) for individual WM fibre populations within each voxel - known as fixels [22]. Given that FBA provides a comprehensive picture of the WM architecture, numerous studies applied FBA to investigate alterations in WM in a wide range of clinical cohorts [23] including multiple sclerosis [24, 25], Alzheimer disease [26], schizophrenia [27], autism spectrum disorder [28, 29], and attention deficit hyperactivity disorder [30] compared to healthy controls. However, to our knowledge, no prior study has utilised these complimentary analytical techniques and advanced metrics to investigate WM alterations in CUD.

To date, only a handful of studies [17, 19, 31, 32] have investigated WM changes in CUD using network-based modelling (e.g., network-based statistics, connectomics). Among significant findings, altered structural connectivity has been observed across the hippocampus, caudate, pallidum [32], cingulate [19], splenium of CC, and right hippocampus [17]. These studies, and their finding, lack the microstructural specificity to fully characterise the integrity of the structural network for several reasons – they did not: (1) used advanced diffusion sequences (e.g., high b-value and high angular resolution) that significantly affect the resolution of the acquired data and is required to resolve for the crossing fibre orientation using constrained spherical deconvolution (CSD) model [33, 34]; (2) employ advanced pre-processing such as outlier replacement [35] and slice to volume motion correction [36]; (3) filter reconstructed WM streamlines to be more biologically plausible [37]; (4) estimate specific measures of WM microstructure such as fibre density and cross-section [22].

As such, further work incorporating advanced diffusion acquisition and pre-processing techniques can help better understand the biological underpinnings of the WM changes in CUD.

In this study, we investigated differences in WM connectivity and microstructure between CUD and healthy controls, using whole-brain connectome and fixel-based analysis. Importantly, we overcome the limitations of previous DTI studies by using the CSD model capturing properties of individual fixels in the presence of crossing fibre bundles [21, 23], which are known to be present in almost 90% of WM voxels [38]. We also assessed potential correlations between WM parameters (i.e., the strength of connectivity between GM nodes, density and cross-section of fibre bundles) and measures of cannabis use, cognition and well-being in CUD. We hypothesized that CUD would be associated with alterations in frontal regions, hippocampus, and corpus callosum compared to healthy controls.

## Materials and methods

### Participants

A total of 98 adults aged between 18–55 were included in the study. Prior to recruitment, we conducted a sample size estimation based on previous studies investigating WM integrity in CUD using a similar experimental design, which reported a medium effect size of 0.46 [17]. To ensure robustness, we chose a more conservative effect size of 0.36 and calculated the required sample size using G*Power [39]. The A Priori analysis indicated that a sample size of 89 would be required to achieve an alpha level of <0.05 and a power of >0.8. The final sample size reported in this manuscript meets this requirement. This sample was comprised of two groups: individuals with cannabis use disorder (CUD, n = 58, median age 25 years, 22% females) and a healthy control group (n = 40, median age 31.5 years, 50% females). The Mini International Neuropsychiatric Interview was used to confirm the presence of moderate to severe CUD and screen for comorbidities [40]. Inclusion criteria for the CUD group was a significant history of cannabis use defined as three or more days of use per week for an average of four of the past six years. Healthy individuals were included in the study if they had used cannabis on less than 20 occasions, and no illicit drug over their lifetime. General exclusion criteria across groups included previous diagnosis of a neurological disorder (such as a head injury) or psychiatric disorder (such as bipolar disorder, obsessive-compulsive disorder, post-traumatic stress disorder, psychosis) or neurodevelopmental disorder (such as autism spectrum disorder); contraindications for MRI (e.g., metal implants or claustrophobia), other chronic medical illnesses (e.g., cardiovascular disease, chronic pain, musculoskeletal injury), and significant or regular use of recreational drugs other than cannabis (moderate or severe substance use disorder based on the MINI). Use of psychotropic medication was assessed, and participants were excluded if taking medication known to impede neuronal plasticity or if medication changed in the four weeks prior to participation. The study was approved by the Monash University Human Research Ethics Committee (Ethics ID #12563, clinical trial registration ID: NCT04902092), and written informed consent was obtained prior to participation.

### Measures

Cannabis consumption was assessed using the Timeline Followback procedure [41, 42], which provided an estimate of the total grams of cannabis each participant consumed across the month (four weeks) preceding the MRI scan [42]. Depression and anxiety symptoms were assessed with the 16-item Quick Inventory of Depressive Symptomatology-Self Report scale (QIDS-SR) [43], and State Trait Anxiety Inventory (both subscales) [44], respectively. Paired associate learning was assessed using the CANTAB Paired Associate Learning (PAL) test [45]. First attempt memory score (FAMS; number of times a participant chose the correct box on their first attempt when recalling the pattern locations) and Total Adjusted Errors (TEA; total errors plus an adjustment for the estimated number of errors they would have made on any trials not completed) were used to assess performance. The Rey Auditory Verbal Learning Test (RAVLT) was used to assess verbal learning and memory [46]. Total learning (total number of items correctly recalled during the learning phase) and recognition (number of items correctly recognised as present or absent from the original list) were used as outcome variables for this task. Clinical and cognitive measures were obtained in person at Monash University. Recruitment was paused between March and November 2020 due to the COVID-19 pandemic and associated lockdowns in Melbourne. These measures were completed online via Zoom once the study recommenced.

Four participants (CUD = 2, Control = 2) were excluded due to missing clinical data and/or having outlier connectivity strength within their connectivity matrix, thus the final analysis was conducted on 56 CUD and 38 healthy control participants.

### MRI analysis

#### MRI acquisition

Participants completed a 45-min MRI scan on a 3T Siemens Skyra at Monash Biomedical Imaging, Monash University. Anatomical T1-weighted images were acquired using a Magnetization Prepared Rapid Acquisition Gradient-Echo (MPRAGE) sequence with parameters: repetition time (TR) = 2300 ms, echo time (TE) = 2.07 ms, 192 slices, 1 mm^3^ isotropic, field of view = 256 mm and the diffusion imaging sequence consisted of 60-diffusion-encoding gradients conducted with TR = 8800 ms, TE = 110 ms, voxel size = 2.5 mm^3^, R»L phase encoding direction. Each diffusion scan acquired 67 volumes (60 volumes with b = 3000 s/mm^2^, and 7 interleaved b0 volumes). L»R direction with b = 0 was also collected for distortion correction.

#### MRI pre-processing

All the image processing performed in the Multi-modal Australian ScienceS Imaging and Visualisation Environment (MASSIVE) high-performance infrastructure [47].

**Fig. 1:**
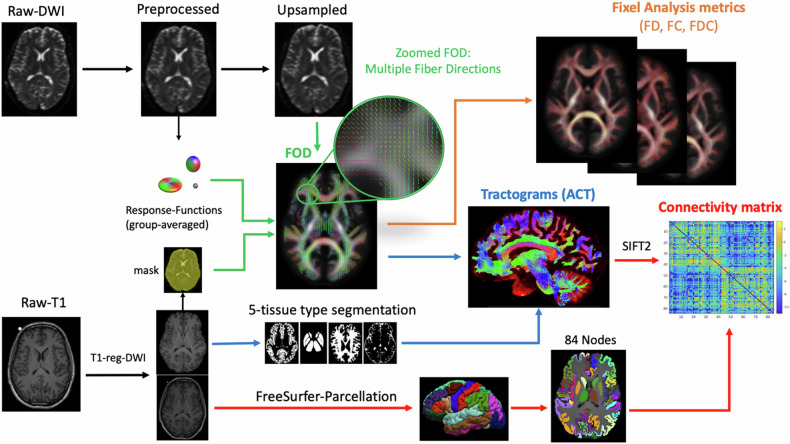
Schematic overview of structural connectome and fixel analysis pipeline.

A schematic overview of the tractography, structural connectome, and fixel analysis pipeline is provided in Fig. 1. In brief, each participant’s anatomical T1-weighted images were pre-processed and parcellated using “recon-all” in FreeSurfer/6.0 (http://surfer.nmr.mgh.harvard.edu/). On this surface model, the FreeSurfer automated cortical parcellation outputs were converted to MRtrix format to generate 84 nodes (cortical grey matter and subcortical regions) using the Desikan Killiany (DK) atlas [48]. Quality of the individual FreeSurfer parcellations was evaluated using the ENIGMA protocol [49]. Diffusion-weighted images were pre-processed using MRtrix3 and FSL software and then upsampled [35, 36, 50]. Individual brain masks generated from T1-weighted images [51] were coregistered to the upsampled diffusion image [52]. White matter fibre orientation distribution (FOD) maps were estimated from pre-processed diffusion images using single-shell 3-tissue constrained spherical deconvolution (SS3T-CSD) guided by averaged group response functions [21, 34, 53], which was followed by global intensity normalisation [54].

Anatomically constrained tractography was performed for each individual by applying the probabilistic (iFOD2) algorithm to the individual’s normalised FOD maps to reconstruct 20 million white matter tracts at the whole-brain level, guided by 5 tissue-type segmented images in MRtrrix3tissue [55]. Spherical deconvolution-informed filtering of tractograms (SIFT2) was applied to obtain biologically plausible reconstructed streamlines [37].

#### Structural connectome

Individual generated tractograms were used to compute symmetric, zero-diagonal structural connectivity matrices (84 × 84), where each value within a matrix represents the sum of streamlines strength connecting a pair of nodes. To normalize the effect of node size, each contribution to the connectome edge was also scaled by the inverse of the volumes of two connected nodes [56].

#### Fixel-based analysis

The fixel-based analysis was performed as previously described in [22, 23, 50]. In brief, normalised FOD images were registered and averaged to yield a FOD template. Individual FOD images were transformed into template space using estimated warps generated during image registration. The WM analysis template fixel mask was generated by segmenting FODs into individual fixels. Fixels within the mask were used to identify the best fixel correspondence across all participants during statistical analysis, using connectivity-based fixel enhancement [57].

Each FOD lobe was then segmented to estimate the fibre density measure (FD), which indicates the number and orientation of fixels in each voxel. To perform group comparison of fixel-wise parameters, the direction of fixels at each voxel was reoriented and assigned to the FOD template based on the Jacobian matrix at the subject level. Fibre cross-section (FC) and the product of fibre density and cross-section (FDC) were also computed. As per the recommendations of MRtrix3 developers, to ensure FC is normally distributed and centred about zero, the log of FC was computed.

### Statistical analysis

For the structural network analysis, the Matlab-based network-based statistic (NBS) toolbox was used to identify altered structural connectivity between CUD and healthy controls at the whole-brain level [58]. A general linear model was applied to independently compare each edge between groups. Due to a significant difference in age between the control and CUD group (*p* = *0.001*) and evidence of sex-based differences in brain morphology [59], these variables were controlled as covariates in all statistical analyses. In brief, a two-sample t-statistic was first calculated for each pair of regions of the DK atlas to test the null hypothesis of equality in the mean value of structural connectivity between groups. Pairs of regions with a t-statistic exceeding a set threshold of 2.9 (reflecting a p-value of 0.005) were systematically searched for any interconnected networks that may yield evidence of a between-group difference. Statistical significance was established via non-parametric permutation testing, with 5000 permutations and a false discovery rate (FDR)-corrected significance level of .05.

For the statistical model of fixel-based analysis, a general linear model was applied to compare group differences in FD, FC and FDC between groups at the whole-brain level, controlling for the effect of age and biological sex [57]. Connectivity-based smoothing statistical analysis was conducted with the connectivity fixel enhancement (CFE) method, using 2 million streamlines. To correct for multiple comparisons, family-wise error-corrected p-values for each fixel were assigned using 5000 non-parametric permutations [57, 60].

Finally, the correlation analysis was used to assess the associations between white matter, cannabis consumption, clinical and cognitive raw scores. Specifically, Pearson correlations between cannabis use (total last month) with depression (QIDS scores), anxiety (both STAI-state and STAI-trait scores), structural connectivity, and white matter microstructure parameters (mean of FD and FDC values) were calculated. All significant imaging findings (i.e., resulting from the significant group differences in network strength, mean of FD and FDC) were also assessed for correlation with PALFAM, PALTEA, and RAVLT learning and recognition scores, age of onset, and age of regular use. Also, correlations between significant findings from two methods (NBS and FBA) were examined to explore if the findings align. Significant findings were defined as *p* < .05 (with Bonferroni correction).

## Results

### Structural network

**Fig. 2:**
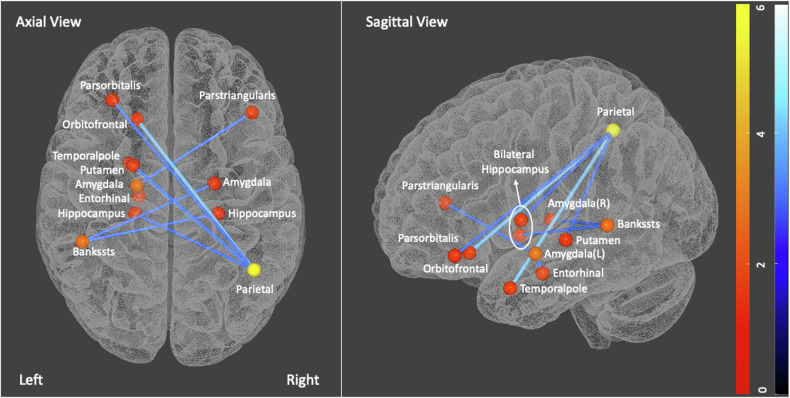
Network-Based Statistics results-structural connectivity differences between CUD and healthy controls. Individuals with CUD showed increased strength across 12 nodes and 9 edges within frontoparietal networks and subcortical regions. The strength of nodes (number of connections with other nodes) is indicated by orange-red colour bar, and edges depicted in blue to dark blue.

We observed significant group differences in white matter connectivity between individuals with CUD and control groups. Compared to healthy controls, those with CUD did not have any statistically significant reduced connectivity (*p* > 0.05) but showed significantly increased strength across 9 edges (*p* = 0.04 FDR corrected) illustrated in Fig. 2. These effects were observed between left entorhinal to left amygdala (T = 3.14), left lateral orbitofrontal to right inferior parietal gyrus (T = 3.6), left pars orbitalis of the frontal gyrus to right inferior parietal gyrus (T = 2.96), left temporal pole to right inferior parietal gyrus (T = 3.65), left putamen to right inferior parietal gyrus (T = 3.37), left hippocampus to right inferior parietal gyrus (T = 3.02), left amygdala to right pars triangularis of the frontal gyrus (T = 3.30), left bank of superior temporal sulcus to right amygdala (T = 3.04), and left bank of superior temporal sulcus to right hippocampus (T = 3.02).

### White matter alterations (FD, FC, and FDC)

**Fig. 3:**
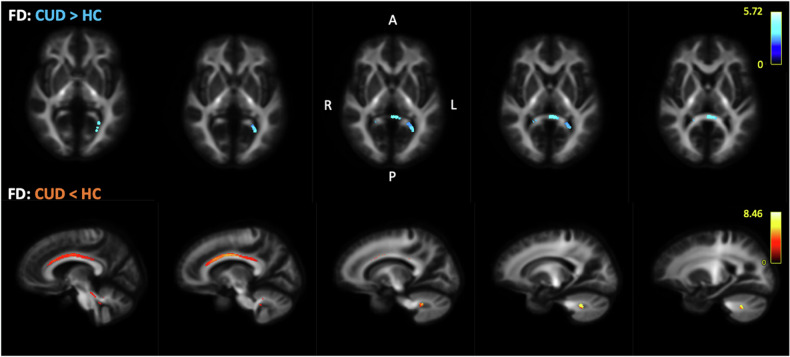
Fixel-Based Analysis: individuals with CUD displayed increased FD in the splenium of the corpus callosum (shown in blue-dark blue, starting slice number 15 with 2 mm increment), and reduced FD within the bilateral cingulum and right cerebellum compared to healthy non-users (shown in yellow-red, starting slice number 36 with 2 mm increment). Scale reflects test statistic values across significant voxels (FWE-corrected, p < 0.05).

**Fig. 4:**
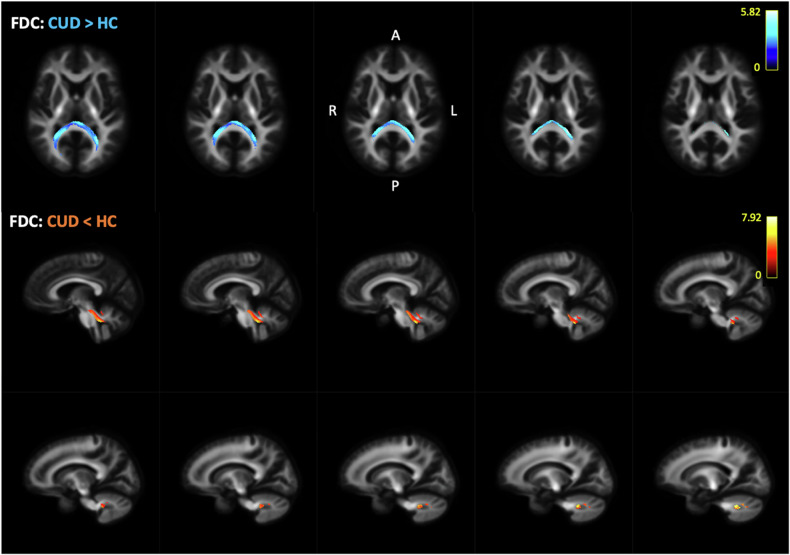
Fixel-Based Analysis: differences in fibre density and cross-section (FDC) values were observed between individuals with CUD and healthy non-users. Participants with CUD had higher FDC across the splenium of the corpus callosum (shown in blue-dark blue, slice number 15 with 2 mm increment), but lower FDC in the right cerebellum (shown in yellow-red, starting slice number 36 with 2 mm increment). Scale reflects test statistic values across significant voxels (FWE-corrected, p < 0.05).

Compared to healthy controls, the CUD group had higher fibre density (FD, microstructure) in the splenium of the corpus callosum, but reduced FD in the bilateral cingulum bundle (across mid-anterior, mid-posterior, and post-dorsal segments) and right cerebellum (middle cerebellar peduncle) (Fig. 3). The effect sizes of FD changes were up to ~50% in the corpus callosum, and up to ~35% in cingulum and cerebellum. No significant group differences observed in the fibre cross-section (FC, macrostructure change). The CUD group showed higher white matter fibre density and cross-section (FDC, the product of FD and FC) in the splenium of the corpus callosum, but lower values in the right cerebellum (Fig. 4).

### Associations among structural connectivity, white matter alteration and cognitive and clinical measures

**Fig. 5:**
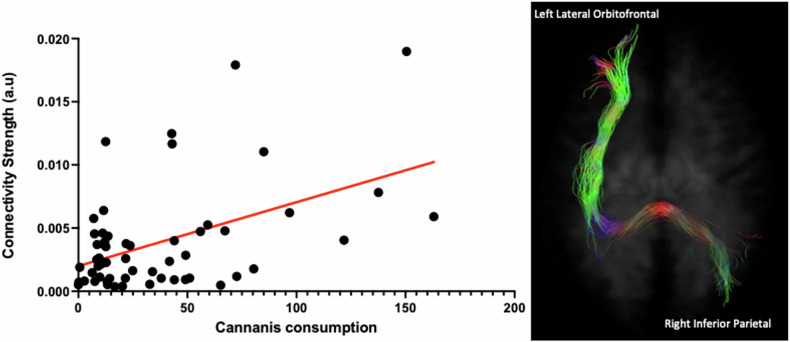
A significant positive correlation was observed between total cannabis consumption (one month prior to study) and connectivity strength between the left lateral orbitofrontal cortex and the right inferior parietal cortex in those with CUD (p = 0.0002). This critical edge encompasses several white matter tracts including left anterior and posterior corona radiata, left anterior and posterior limb of the internal capsule, left external capsule, splenium of the corpus callosum, and right cingulum.

We did not observe any significant association between WM, cognitive and well-being measurements (*p* > 0.05). However, higher self-reported total cannabis consumption across the previous month was associated with higher structural connectivity between the left lateral orbitofrontal cortex and the right inferior parietal lobe (*p* = 0.00020, *r* = 0.47). According to the JHU (Johns Hopkins University) atlas, the streamlines between these two GM regions pass through several WM tracts including the splenium of the corpus callosum, inferior longitudinal fasciculus (left), anterior corona radiata (left), posterior corona radiata (bilateral), anterior and posterior limb of internal capsule (left), external capsule (left), and cingulum (right) illustrated in Fig. 5 [61]. Also, the mean of FD values within the bilateral cingulum and right cerebellum was positively associated with the age at which regular cannabis use was commenced (*p* = 0.0066, *r* = 0.36), but not the onset age of recreational use. In other words, those who initiated chronic use of cannabis at younger ages experienced the greatest WM density reduction across these tracts. Regarding the exploratory analysis, higher structural connectivity between the left lateral orbitofrontal cortex and the right inferior parietal lobe was associated with higher FDC in the corpus callosum (*p* = 0.01, *r* = 0.32). Similarly, higher connectivity between the left putamen to right inferior parietal was correlated with higher FD in the corpus callosum (*p* = 0.023, *r* = 0.30). No other significant associations were observed.

## Discussion

In this study, we investigated structural connectivity and white matter alterations between chronic cannabis users and healthy non-using controls via whole-brain network statistics and fixel-based modelling. We observed higher structural connectivity strength in those with CUD relative to controls across the frontoparietal network, and between the right inferior parietal cortex and subcortical regions including left hippocampus, left entorhinal and left putamen. Although we observed altered white matter density in the splenium of the corpus callosum, right cerebellum, and bilateral cingulum in those with CUD, there were no significant differences in cross-sectional area of these white matter bundles. This implies that CUD may have an impact on the microstructural properties of white matter rather than its macrostructure. Overall, our results indicate that CUD is associated with significant and widespread differences in brain white matter architecture compared to healthy controls.

Our findings broadly align with network-level connectivity studies demonstrating altered cortico-subcortical connectivity in CUD. For example, previous studies have reported altered connectivity across the hippocampus, caudate and pallidum [32], and higher segregation in frontoparietal regions [31]. Similarly, one connectome study also reported reduced connectivity between the hippocampus and splenium of the corpus callosum [17]. Furthermore, our results from structural network analysis demonstrated the right inferior parietal lobe as a critical hub (having the highest number of connections with other nodes, 5 out of 9 altered edges). This is a vital neural junction that supports both fundamental and complex mental processes from attention to memory and social cognition [62], highlighting the significance of related WM pathways for neural communication to this region. We did not observe evidence of associations between WM metrics and any cognitive or mental health outcomes, which is in line with the recent systemic review by Robinson et al. [63]. However, we observed a significant association between total cannabis consumption (the last month prior to the study) and the strength of connectivity between the left lateral orbitofrontal cortex and the right inferior parietal lobe. This edge encompasses several critical WM tracts including the corpus callosum, inferior longitudinal fasciculus, internal and external capsule, and anterior and posterior corona radiata. Alterations in these WM tracts have been associated with chronic cannabis use [63].

Regarding the white matter microstructure, we provide the first fixel-based evidence of altered fibre density of white matter bundles in CUD. Considering that CUD-induced structural effects are subtle, it is crucial to employ advanced methods specific to WM properties to accurately investigate pathological changes to the WM microstructure. Since previous literature suggested that the DTI model could lead to misleading findings [26, 27], numerous WM studies on different clinical cohorts have applied the FBA method to investigate subtle pathological changes in WM microstructure by providing more biologically meaningful measurements [23]. Previous FBA literature has consistently reported significant reduction of FBA metrics in patients with neurological diseases [24–27, 64], as well as neurodevelopmental disorders such as autism spectrum disorder [28, 29], and attention deficit hyperactivity disorder [30] compared to healthy controls. In contrast, however, increased FBA measurements were observed in patients with social anxiety disorder [65] which aligns with the direction of change we observed.

To ensure accurate interpretation of the results, we used high-quality diffusion acquisition parameters to minimize contamination from extracellular space signals in the observed changes [23]. As such, the higher FD we observed is indicating greater axonal volume (or stronger structural connectivity due to higher streamlines) in the corpus callosum in CUD (and not myelinogenesis) and lower FD values imply reduced axonal volume in the cingulum and right cerebellum and not necessarily demyelination along these fixels compared to controls. To further enhance our understanding of the FBA results, our correlation analysis showed WM fibre density in the cingulum and right cerebellum was correlated with age of regular cannabis use, suggesting that individuals who regularly consume cannabis from a younger age may be at greater risk of reduced WM density in these regions. This is consistent with previous diffusion literature showing that reduced WM microstructure is associated with younger age of cannabis onset [63]. Moreover, previous cannabis studies demonstrated significant association between early cannabis onset and decreased regional brain volume (e.g., hippocampus and amygdala) as well as altered functional connectivity [2]. These findings may reflect the influence of cannabis exposure on brain maturation, possibly through the disruption of the endocannabinoid system, however, the exact mechanisms underlying these associations remain unclear [63].

While structural network analysis has been employed in a limited number of studies, other imaging modalities have consistently shown changes in similar networks in those with CUD. For instance, several functional imaging studies have reported altered connectivity across frontal, parietal, and cingulate regions among adult cannabis users [14, 66, 67]. Together, the altered brain regions we identified herein overlap with those significantly affected regions reported in the fMRI literatures, including frontoparietal networks (e.g., decision-making, inhibitory control) [68, 69], striatal circuits (e.g., reward processing, emotional regulation) [70, 71], and medial temporal regions (e.g., memory and learning) [72]. Moreover, many of these observed alterations express a high density of endocannabinoid CB1 [2], and/or inhibitory amino-acid aminobutyric acid (GABAergic) receptors [73] indicating that regions with a high affinity for cannabinoids may be more susceptible to the structural changes observed herein. Notably, CB1 receptors are highly concentrated in mesolimbic circuits including the hippocampus, amygdala and striatum which prominently project into cortical regions such as the prefrontal cortex, orbitofrontal, and cingulate [74]. Beyond the well-known neuroimaging findings showing that CUD affects the volume of the brain regions within this circuit [3], our results provide additional evidence that CUD is associated with disruptions in the underlying white matter pathways connecting this circuit. For instance, literature has suggested that THC may introduce WM alterations around these regions rich in CB1 receptors, which consequently may disturb neuronal communication across the circuit [75, 76]. Further, considering the biphasic effects of cannabinoids, chronic exposure to cannabis may induce a neuroinflammatory response via pro-inflammatory cytokines [77], potentially leading to further WM impairment.

By elucidating the precise mechanisms of microstructural differences in individuals with CUD, we aim to provide a clearer understanding of how CUD impacts white matter microstructure. At this point, it is unclear if these differences exist prior to CUD or manifest because of CUD. Our findings of a dose-related association between WM integrity and cannabis exposure would support the latter. Future longitudinal studies using advanced diffusion methods are needed to better understand the specific temporal relationship between CUD and these WM microstructure changes, and the potential for interventions to reverse them. If indeed these alterations pre-exist exposure to cannabis, they may be markers of risk for CUD. On the other hand, if these WM alterations are causally linked to chronic exposure to cannabis, then it would be important to develop screening tools to identify individuals who are at the highest risk of developing these changes (e.g., early onset users, those of a certain demographic, etc.) and provide educational information about the risks of CUD and benefits of abstinence (e.g., recover of cognitive impairment) [78, 79]. Regular physical exercise has been shown to significantly improve WM health [80, 81], which highlight its potential as non-pharmacological intervention to recover WM impairment in CUD. It is also possible that the observed WM alterations may be associated with certain profiles of cannabis (e.g., high THC – low CBD), in which case there is room to inform policy to regulate certain types of cannabis deemed to be most harmful [74, 82].

## Conclusion

To our knowledge, this is the first study applying complementary network and fixel-based analysis to concurrently assess micro and macro-scale white matter alterations in those with CUD. We provide evidence of altered frontoparietal, and striatal networks implicated in executive function and reward processing that may underpin some of the mental health and social function disruptions seen in these individuals. Future research may consider how these network changes contribute to the functional and behavioural symptoms associated with the disorder.

## Data Availability

The original data is available from the corresponding author upon request.
